# The Effect of Ultrasonic Water Treatment on the Change in the Microstructure of Wheat Grain, Dough, and Wheat Flour Bread

**DOI:** 10.1155/2022/1986438

**Published:** 2022-01-29

**Authors:** Natalia Naumenko, Irina Potoroko, Irina Kalinina, Ekaterina Naumenko, Eva Ivanisova

**Affiliations:** ^1^South Ural State University (National Research University), 76 Lenin Avenue, Chelyabinsk 454080, Russia; ^2^Slovak University of Agriculture in Nitra, Tr. A. Hlinku 2, 949 76 Nitra, Slovakia

## Abstract

Visualization of the microstructure of the food matrix of both raw materials and the final product is one of the keys to understanding the processes occurring during its formation. It is the fixation of the results at the microlevel that allows us to form a hypothesis and then confirm it with the obtained array of experimental data. The presented study is aimed at studying the effect of ultrasonic water treatment on the change in the microstructure of wheat grain during its humidification. The article also presents the results of studying the microstructure of dough and wheat flour bread obtained using water after ultrasonic water treatment and the intensity of the processes of staling of finished bread in storage. The object of the study was grain of soft spring white wheat (*Triticum aestivum L.*), varieties of Lubava, harvest 2014-2018, Russia (the protein content was 12.5 ± 0.3 g/100 g in terms of humidity); dough and bread made from wheat flour (ash content 0.55%, mass fraction of gluten 28.5%), produced using the technology of plain bread, a classic recipe without improvers. Ultrasound-treated water with an exposure frequency of 22 ± 1.65 kHz and with a power variation of 252-630 W/l was used in test technology. The experimental data obtained made it possible to establish the intensification of the processes of swelling of wheat grain during soaking. In the experimental samples, after 8 hours of soaking, the loosened structure of the endosperm and evenly swollen components of the grain were observed, and the loop of the groove was closed. Activation of the processes of dough science was established, and gluten flour in the dough formed a single monolithic frame, in which the swollen starch grains are tightly packed. The interstitial walls of the crumb of the prototypes consisted of a solid mass of protein coagulated during baking, inside of which swollen gelatinized starch grains are interspersed, they are closely adjacent to the mass of coagulated protein with their entire surface, and therefore, there is no sharp, clearly visible boundary between them. The most pronounced changes in the structure of the dough and bread crumb were noted when using water, after ultrasonic water treatment at a power of 504 and 630 W/l. This method of exposure can be recommended as the best for obtaining good quality bread with less pronounced staling during storage.

## 1. Introduction

Numerous studies in recent years, in the field of ultrasonic exposure in the frequency range from 20 to 100 kHz, in the production of food products, prove its high effectiveness on the processes of formation of the food matrix. The mechanisms of ultrasonic exposure of various intensities allow you to adjust the technological parameters to intensify the production processes, improve the properties of the product, and increase the preservation of its properties during storage [1–5].

Ultrasonic exposure, as a new technology of nonthermal treatment, due to such effects as cavitation, dispersing, and thermal, can be used to increase the production of biologically active compounds, including primary and secondary metabolites in plant-based foods [6, 7]. The use of sonochemistry processes in food technology caused by the formation and subsequent collapse of an air bubble due to the internal pressure in it (up to 2000 kPa) determines multiple effects in the food matrix system of the product [1, 2, 8]. The grea dough effect of ultrasonic exposure is observed in changing the microstructure of the matrix of food raw materials and the finished product. This result can manifest itself not only when processing the entire matrix of the food system but also when acting on its individual components, for example, on water. Using ultrasonic exposure as a technological operation at the stage of water treatment, it is possible to obtain significant results characterizing an increase in the permeability of the membranes of the shell cells of wheat grain, the development of a protein matrix, and the swelling of starch grains of wheat dough [9, 10]. Thus, we supposed that the ultrasonic exposure is one of the environmentally friendly technologies [11].

The mechanism of ultrasonic exposure is described in the works of several authors [12, 13]. They note that ultrasonic vibrations cause the formation, growth, and heavy destruction of microbubbles in the treated liquid medium. Ultrasonic cavitation in liquid media alternately compresses and stretches the molecular structure of the medium. The fluid medium can literally rupture, forming tiny cavities (microbubbles) during each stretching (rarefaction) phase. These cavities can heavily collapse at the stage of exposure, releasing large amounts of energy in the immediate proximity of the microbubbles. The mechanical and chemical effects of collapse occur in two different areas: (i) inside the bubble with high pressure and a sharp rise in temperature and (ii) in the immediate proximity of the bubble, where a shock wave generated by the collapse creates enormous shear forces that change the physical and chemical properties of the water. The shock waves break hydrogen bonds in the water, which is similar to what occurs when the water is heated to the boiling point [10, 11]. The high adsorption activity of hydroxyl ions formed under ultrasonic exposure causes accelerated wet-out of biological objects. The method of electron scanning microscopy for studying the effect of ultrasonic water treatment on the change in the microstructure of wheat grain, dough, and wheat flour bread is the most effective method in forming the evidence base of the effects of sonochemistry.

Kim et al. [14] investigated the various stages of baking bread using SEM. After the fermentation process, the structure of the protein lattice had large air cells, and fibrils were visualized. The resulting bread had thin walls, and the protein stretched on starch grains was visualized as swollen and melted. The obtained data allowed us to conclude that many small starch granules remain intact and the baking process does not radically change the protein ultrastructure.

Şimşek [15] compared the characteristics of developed and undeveloped wheat dough at different mixing intensity. In their publications, they noted that gluten in an undeveloped dough has an inermittent mesh structure in contrast to the continuous membrane-like structure of gluten in a developed fermented dough.

Li et al. [16] used SEM to study the microstructure of white bread. They reported that the way the samples were prepared greatly affected the structure of the bread crumb.

The above confirms the relevance and practical significance of using SEM to study the microstructure of the matrix of food products. Nevertheless, studies combining the effect of ultrasonic exposure in the process of water treatment on changes in the microstructure of wheat grain, dough, and wheat flour bread are still not well studied and presented in the literature and are of scientific interest.

The aim of the study was to study the effect of ultrasonic water treatment with a power variation of 252–630 W/l on the change in the microstructure of wheat grain, dough, and wheat flour bread.

## 2. Materials and Methods

### 2.1. Materials

This study used a grain of soft spring whitegrain wheat (*Triticum aestivum L.*), a variety of Lubava, harvested in 2014-2018, grown in the Ural region, Russia. The protein content was 12.5 ± 0.3 g/100 g in terms of humidity (Sample 1).

The grain of soft winter whitegrain wheat (*Triticum aestivum L.*), a variety of Erythrosperium 59, harvested in 2014-2018, grown in the Ural region, Russia. The protein content was 12.2 ± 0 : 3 g/100 g in terms of humidity (Sample 2).

The ingredients used to make the bread were purchased at a market in the city of Chelyabinsk. Refined wheat flour (RF) (gluten 29.9%, ash content 0.55) was provided by the manufacturer OOO Soyuzpishcheprom, Chelyabinsk, Russia, and was used as the main raw material in the manufacture of bakery products.

Yeast and salt were purchased in the retail network of the city of Chelyabinsk (pressed baking yeast Lux extra, manufacturer of OOO SAF-NEVA, food salt of the highest grade, manufacturer of OOO Russol).

We used USTA-0.63/22 OM (Volna, Russia) as an ultrasound generator with piezoelectric oscillation system in a metal frame with forced cooling (oscillation frequency was 22 ± 1.65 kHz and intensity range was 252–630 W/l). 4 power modes were used for processing: 252, 378, 504, and 630 W/l (40, 60, 80, and 100% of the power of the device from the passport value). The water was treated with ultrasonic exposure (ultrasound) with each power for 5 minutes; it was used for soaking wheat grains, kneading dough to make bread. Soaked wheat grain, dough, and bread samples obtained using drinking water were used as a control sample.

The results of studies by the authors [12, 13] enabled us to apply these modes of ultrasonic exposure to evaluate the sonochemical reactions in the interaction with the wheat grain matrix, or in the dough and bread formation. The power range of ultrasonic exposure (252-630 W/l) was specified taking into account [11] the fact that the maximum yield of cavitation (sonochemical oxidation and oxidative radicals generating) was observed in moderate power conditions, approximately 400 W/l. This power range was aimed to evaluate the cavitation effect on changes in microstructure: (i) wheat grain during soaking, (ii) bread dough, and (iii) bread crumb after 3 and 72 hours of storage.

### 2.2. Scanning Electron Microscopy (SEM) of the Dough

Electron scanning microscopy of wheat grain after soaking was carried out using a high-resolution scanning electron microscope with a Schottky cathode TESCAN MIRA3, equipped with a nitrogen-free Ultim Max 100 microanalysis system, controlled by Aztex Energy software. Preprepared sections of the studied samples with graphite deposition and a magnification of 150x, 2000x, and 3000x.

The dough was prepared according to the method of Kim et al. [4, 17], with some modifications, using Farinograph equipment. The dough and bread samples were dried on freeze-drying equipment (Coolvacuum Technologies Lyomicron-55C, Spain), with subsequent spraying of platinum. To obtain micrographs, a scanning electron microscope (JSM-7001F (JEOL), Japan) was used at 20 kV and a magnification of 1000x. The dough swelled for 120 minutes before microscopy.

### 2.3. Soaking of Wheat Grain (*Triticum aestivum L.*)

Soaking of wheat grain was carried out for 8 hours, in a hydromodule grain: water 1 : 1.25, at water temperature 20 ± 2°C [15]. For soaking, drinking water (control) and water treated with ultrasonic power of 252, 378, 504, and 630 W/l were used, respectively, samples 1 (a, b, c, and d) and samples 2 (f (252 W/l), g (630 W/l).

### 2.4. Test Laboratory Baking

The test laboratory baking was carried out using the international method AACC 10-10.03 [18]. The recipe of the obtained samples is presented in Table 1 [10].

The amount of water was calculated based on the water absorption capacity of the flour at the rate of 650 E.F. (± 20) on the Brabender Farinograph equipment. The water temperature was 22 ± 2°C. The rheological properties dough were determined in accordance with the AACC 54-21.02 (2010) method on a Farinograph-AT (Brabender, Germany). The volume of bread was determined by the seed displacement according to the method AACC 10-05.01, 2010. The bread was weighed, and its specific volume was calculated. The moisture content of the bread crumb was determined according to AACCI 44-15.02, 2010.

The dough was divided by hand into portions of 450 g, and fermentation was carried out in a controlled fermentation chamber (Atrepan 18/10, Italy), at 30 ± 1°C and a relative humidity of 80% for 90 minutes. Baking was carried out in a laboratory oven (Fin Bake II 5 D Digital, Slovenia) at 220°C for 20 minutes. The bread was cooled at 20 ± 2°C and tested after 3 and 72 hours.

### 2.5. Statistical Processing of Results

The studies were conducted in a fivefold repetition. The experimental data were processed on the basis of mathematical statistics methods using Microsoft Excel and MathCad. The obtained data are presented with a confidence factor of 0.95.

## 3. Results and Discussion

### 3.1. The Effect of Ultrasonic Water Treatment on the Change in the Microstructure of Wheat Grain

To establish the effect of ultrasonic exposure on the changes occurring in the grain during its soaking, a study of microstructural changes in experimental samples of wheat grain was conducted. Considering the nature of water absorption by structural elements of wheat grain, described in the works of Miller and his colleagues [19], and the sequence of filling its structural elements with moisture, it is very significant, using modern optical equipment, to study the effect of ultrasonic water treatment on the process of moistening the longitudinal, transverse and tubular layers of fruit shells, pigment and hyaline layers of seed shells, endosperm, and embryo.

**Figure 1:**
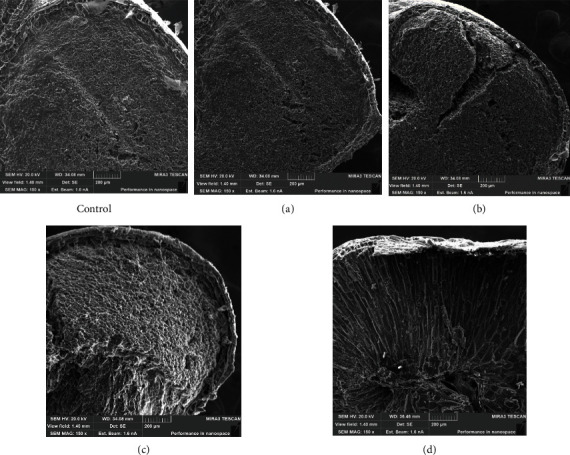
Characteristic view of electronic microphotographs of the cross-section of the control and experimental (a–d) wheat grain samples (Sample 1) after 8 hours of soaking, TESCAN MIRA3 SEM, magnification 150x.

The study of microstructural changes in control and experimental samples of wheat grain (Sample 1) was carried out after 8 hours of the soaking process, which is due to the achievement of the nominal value of the swelling rate indicator. Analyzing the results of SEM (Figure 1) of cross-sections of wheat grain samples, one can observe their obvious difference.

Microphotographs of cross-sections of the control sample (Figure 1 (control)) visualize the densely packed structure of the endosperm, with a smaller magnification, a groove is noted, which indicates the initial stages of the swelling process. The microstructure of sample a (Figure 1(a)) has the greatest similarity with the control sample; tears of no more than 200 *μ*m are noted in some areas. When using ultrasonic-treated water with a capacity of 378 W/l (Figure 1(b)) for soaking, activation of this process is noted, and individual large tears of the gluten and starch matrix with a size of more than 500 *μ*m are visualized. A single swollen system of gluten and starch grains is observed when using water, treated with ultrasound, with a capacity of 504 W/l (Figure 1(c)), there is swelling of the seed shells, pulling gluten into separate hyphae. In the sample Figure 1(d)), the loosened structure of the endosperm and evenly swollen components of the grain are visible, with a smaller magnification; it is visualized that the loop of the groove is closed. This characterizes an intensive swelling process, probably due to an increase in the permeability of cell membranes due to the use of ultrasound-treated water with a capacity of 630 W/l in the process.

**Figure 2:**
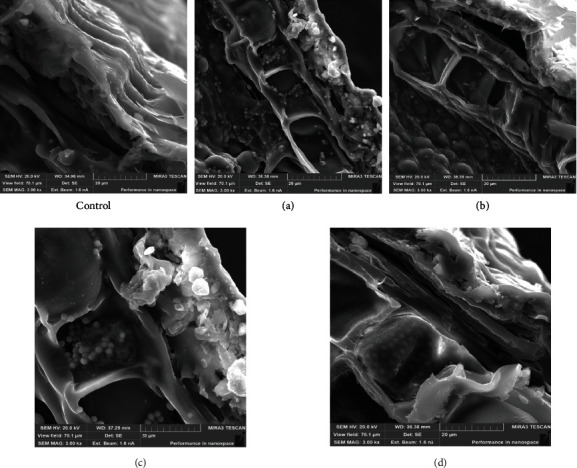
Characteristic view of electronic microphotographs of the cross-section of the control and experimental (a–d) wheat grain samples (Sample 1) after 8 hours of soaking, TESCAN MIRA3 SEM, magnification 3000x.

A detailed study (Figure 2) of the fruit and seed shells of control and experimental samples of wheat grain after 8 hours of soaking proves differences in their condition. The fruit and seed shells of wheat grains in two experimental samples (c) and (d) are swollen after the soaking process, the stratified transverse and longitudinal layers of the fruit shell, the swollen hyaline layer of the seed shell with longitudinal elongated cells are distinguished, and the aleurone layer is also well visualized, which is explained by the increased permeability of cell membranes [20].

**Figure 3:**
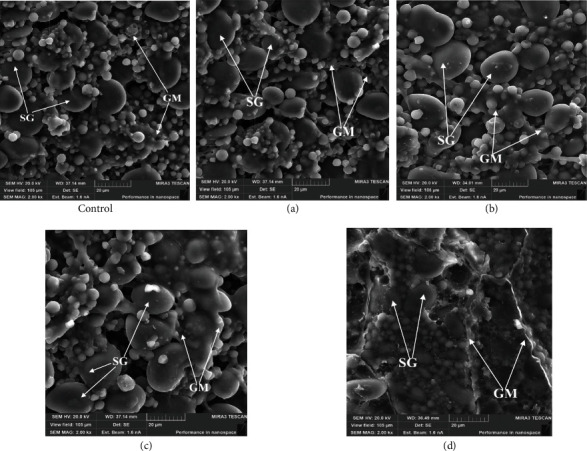
Characteristic view of electronic microphotographs of the cross section of the control (a) and experimental (b) wheat grain samples (Sample 1) after 8 hours of soaking, TESCAN MIRA3 SEM, magnification 2000x: SG: starch grains; GM: gluten matrix.

Ultrasonic waves during water treatment exhibit the effect of cavitation in the form of vibrations of vapor-gas bubbles and their collapse, which changes the physicochemical properties of water. Under the influence of shock waves of the cavitation field, hydrogen bonds break in the structure of water, similar to what happens when it is heated to the boiling point [2, 21]. The high adsorption activity of hydroxyl ions formed during ultrasound causes accelerated wetting, which is confirmed by the data obtained in studies of wheat grain samples. The study of the state of the endosperm after soaking using SEM at an increase of 2000x (Figure 3) makes it possible to fix differences in the microstructure of the endosperm of control and experimental wheat grain samples.

In the endosperm of the control sample (Figure 3 (control)), starch grains of various sizes are visualized; the gluten matrix in the form of separate threads envelops them only partially. The presence of mainly (70%) small and medium starch grains is noted. The microstructure of sample a (Figure 3(a)) has minor differences from the control, it is also characterized by the presence of a large number of non-swollen starch grains, and the gluten matrix is visualized as separate fibrils partially covering the grains. When using water treated with ultrasound, with a capacity of 504 W/l (Figure 3(c)), there is an intensive development of a gluten matrix that completely covers starch grains. The developed protein matrix has a single monolithic structure; thick swollen gluten fibrils are visualized in some segments.

In the microstructure of the sample Figure 3(d)), there is a large amount of swollen gluten, which completely envelops the starch grains. They have different sizes and are well embedded in the protein matrix. Some studies have found that water is able to weaken the connection of the gluten–starch system [22]; in the future, it can have a positive effect on the process of grinding wheat grains and minimize the number of damaged starch grains.

**Figure 4:**
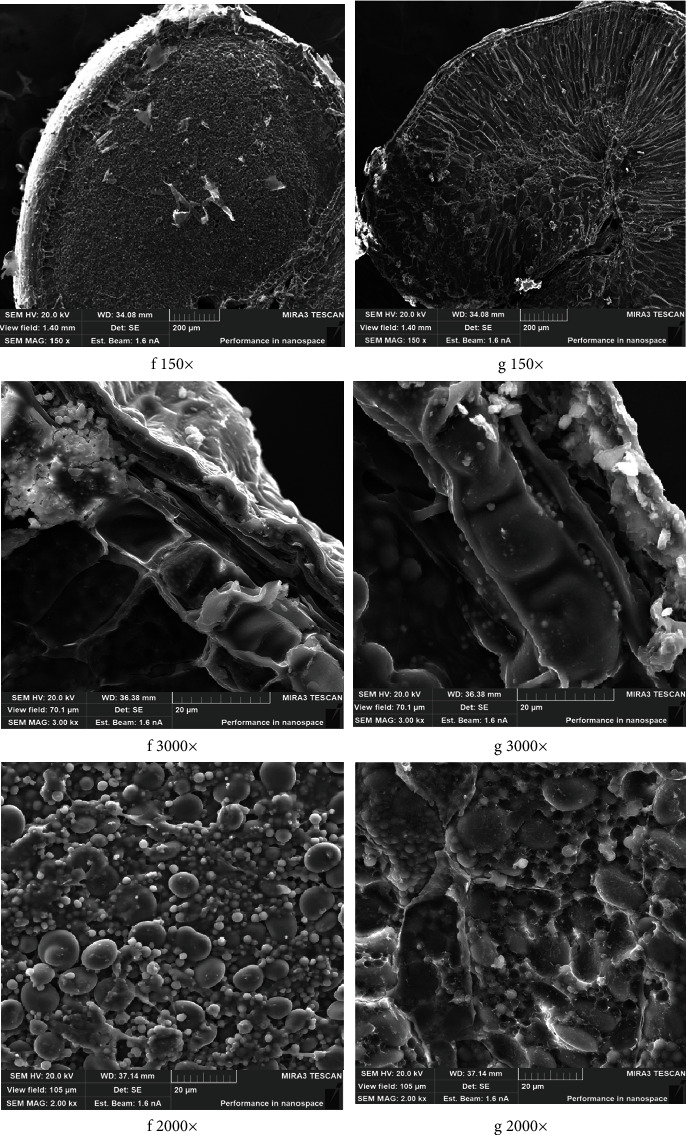
Characteristic view of electronic microphotographs of the cross-section wheat grain samples (Sample 2) after 8 hours of soaking, TESCAN MIRA3 SEM, magnification 150x, 3000x, and 2000x.

The obtained effects were also proved when examining the microstructure of winter white wheat (Sample 2); the results of which are given in Figure 4.

The microstructure of sample f (Figure 4(f)) has a large number of nonswollen starch grains; the gluten matrix is visualized as individual fibrils partially covering the grains. With water treated with 630 W/l UHV (Figure 4(g)), a large amount of swollen gluten completely envelopes the starch grains of different sizes. The enveloped parts swelled well while soaking. The presented results suggest that mechanical or shear effects from the acoustic vibrations and bubble sizes of the water treated with UHV resulted in grain soaking intensification, which improves permeability of the plant cell walls and thus increases water absorption by the enveloped parts.

It is known that the swelling of wheat grain is accompanied by a number of physiological processes: the respiratory activity of the grain is resumed, and protein synthesis from existing RNA occurs [18]. When RNA is restored, which was damaged during the drying phase of wheat grain, the functions of all cellular organelles, in particular mitochondria, begin to manifest [21]. Consequently, in case of ultrasound, by increasing the permeability of the cell membranes of the fruit and seed shells of wheat grain, the process of moisture migration from the outer to the inner parts of the grain is accelerated during soaking. This process is most pronounced when using ultrasonic-treated water with a capacity of 630 W/l [22].

### 3.2. The Effect of Ultrasonic Water Treatment on the Change in the Microstructure of Wheat Dough

**Figure 5:**
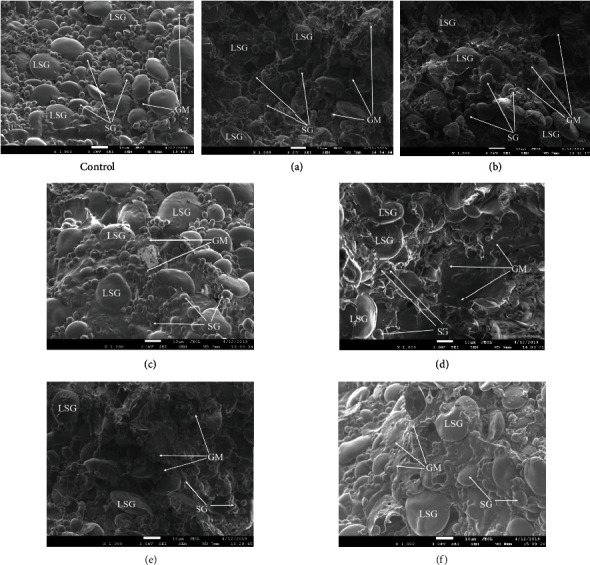
Results of the study of the microstructure of the dough (SEM) after 120 minutes of fermentation. LSG: large starch grains; SG: small- and medium-sized starch grains; GM: gluten matrix.

The use of water treated with ultrasound also has a significant impact on the microstructure of wheat dough (Figure 5).

To study the microstructure of dough samples after fermentation for 120 minutes, they were lyophilized and analyzed with preliminary platinum spraying in vacuum, recorded with an electron microscope of JSM-7001F (JEOL).

The structure of both control and experimental (a, b, c, and d) dough samples (Figure 5) is characterized by the presence of a large number of oval-shaped particles ranging in size from 5 to 30 microns, which in their characteristics correspond to starch grains. The surface of the grains is smooth, without cracks, grooves, and pores. It is possible to distinguish the presence of large, medium, and small starch grains, the ratio of which varies depending on the used power of the ultrasound. Starch granules are clearly distinguishable, have a spherical shape, are visualized on the surface, and have a similar structure [23].

In the control sample (Figure 5 (control)), more small- and medium-sized starch granules are visualized. Starch is present in the form of round or elliptical shaped grains. Individual grains are slightly deformed; this is more often observed in large starch grains. Individual starch grains have attached gluten particles, which gives them angularity.

The greatest differences are visualized in experimental samples obtained using water treated with ultrasound, with a capacity of 504 and 630 W/l (Figures 5(c) and 5(d)), the presence of starch grains having a biconvex shape is noted. Swollen, significantly increased in size, large starch grains with a size of 20 to 30 microns predominate.

The gluten matrix is developed in all samples somewhat differently. In the control sample (Figure 5 (control)), as well as in the experimental (Figures 5(a) and 5(b)), it is insufficiently developed; only in rare cases several grains of medium and fine starch are covered with a solid layer of protein. The structure is quite loose, as there are a large number of air cavities. In some cases, it is possible to consider the structure of an intermittent gluten matrix, which has the form of appendages connecting starch grains. Free gluten globules are not visible in the photos.

In the experimental samples (Figures 5(c) and 5(d)), the gluten matrix is more clearly distinguishable and evenly distributed and surrounds most of the starch grains, combining them. Numerous holes from the formed air bubbles are visualized on the surface of the protein matrix, which indirectly confirms the more intensive fermentation process of the tesa. Experimental samples obtained with winter wheat flour (Figures 5(f) and 5(g)) also prove the relationships described above. The gluten matrix of the sample (Figure 5(g)) is abundantly developed and completely envelopes both small and large grains of starch.

Based on the above, it is possible to formulate a hypothesis that the use of water treated with ultrasonic power of 504 and 630 W/l in the formulation eliminates the energy costs of water penetration into the starch and gluten molecule, which allows it to penetrate more easily and faster into the matrix, which contributes to a good water absorption capacity of flour, the formation of the dough frame, and an increase in the yield of products.

### 3.3. The Effect of Ultrasonic Water Treatment on the Change in the Microstructure of Wheat Flour Bread

**Figure 6:**
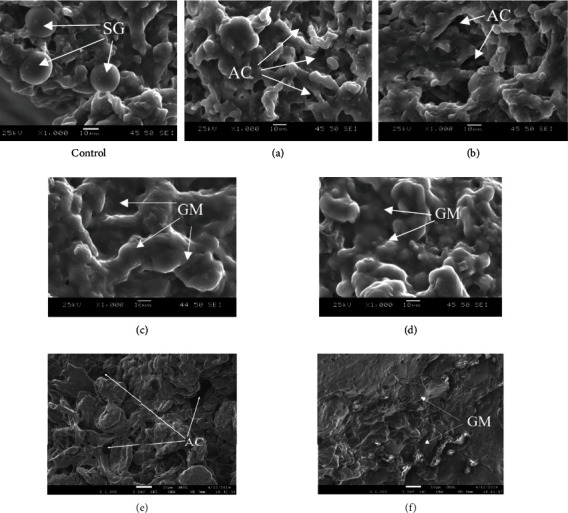
The microstructure of the bread crumb after 3 hours storage: SG: starch grains; GM: gluten matrix; AC: air cavity.

The microstructure of the bread crumb after 3 hours storage is presented in Figure 6.

In control bread samples (Figure 6(a)), we can visualise separate large and medium starch grains (SG) not incorporated in the gluten matrix. The samples obtained using treated water at 252 W/l and 378 W/l intensity have more air cavities (AC), partially coagulated gluten skeleton, with starch grains. The crumb of the bread samples obtained using treated water at 504 W/l and 630 W/l intensity have a uniform mass of coagulated during baking gluten, which incorporates swollen gelatinized starch grains which are firmly attached to the gluten matrix (GM). The ultrasonic water treatment due to cavitation effects increases penetration into the product matrix, when exposed to 504 and 630 W/l. Thus, the gluten matrix is developed, which covers the entire surface of the starch grains. This result improves the consumer properties of the finished samples of bread.

The influence of ultrasonic water treatment due to cavitation effects on the quality of bread was evaluated by the specific volume and humidity (Table 2). The use of ultrasonic water treatment increased the specific volume of bread. Ultrasonic treatment of water when exposed to 504 W/l (c) increased the specific volume of bread by 10% relative to the control sample, and treatment with 630 W/l—by 15%. The moisture content of all bread samples ranged from 40.2 to 41.6%.

**Figure 7:**
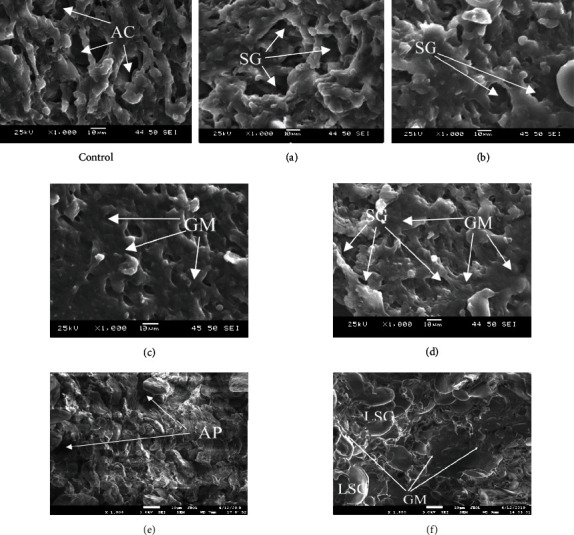
The microstructure of the bread crumb after 72 hours storage: GM: gluten matrix; AC: air cavity; AP: air pores.

It is common knowledge that staling determines the changes in the microstructure of the crumb, while cooling and further storage due to the flexibility of starch chains the chains get converged and molecular van der Waals forces form a mechanically strong grid [18, 22]. The formation of the grid leads to the staling of the crumb when the mechanical density of the structure reaches maximum level. The results of the study obtained using scanning electron microscopy of bread samples after 72 hours of storage (Figure 7).

The observance of the changes of the microstructural indicators in control and experimental bread samples after 72 hours demonstrate the following:

- Air layers (AC) are clearly visible in the microstructure of the control sample, which may indicate a decrease in the volume of starch grains due to the formation of the starch crystal structure
- Experimental samples obtained using treated water at 252 W/l and 378 W/l have a more uniform amorphous crumb structure, with a smaller number of air spaces. However, it is possible to distinguish starch granules in the samples and determine the pore size (AP)

The most pronounced differences are characteristic of the sample obtained on water treated with ultrasound, with a capacity of 504 W/l (Figure 7(c)). Its crumb structure can still be viewed as a swollen, structureless gel with subtle pore walls. For a sample obtained on when using water treated with ultrasound, with a capacity of 630 W/l (Figures 7(d) and 7(g)), a large mass is represented by a swollen amorphous structure (GM), but there are isolated air pores (AP).

## 4. Conclusions

Ultrasonic exposure (25 kHz) used in this study can be one of the effective environmental factors that stimulate the process of soaking wheat grains, forming dough and bread and wheat flour. The experimental data obtained made it possible to establish the intensification of the processes of swelling of wheat grain during soaking. In the experimental samples, after 8 hours of soaking, the loosened structure of the endosperm and evenly swollen components of the grain were observed; the loop of the groove was closed. Activation of the processes of dough science was established; gluten flour in the dough formed a single monolithic frame, in which the swollen starch grains are tightly packed. The interstitial walls of the crumb of the prototypes consisted of a solid mass of protein coagulated during baking, inside of which swollen gelatinized starch grains are interspersed, they are closely adjacent to the mass of coagulated protein with their entire surface, and therefore there is no sharp, clearly visible boundary between them. The greatest effect in changing the microstructure was established when using water treated with ultrasonic power of 504 and 630 W/l. Considering the importance and relevance of this study for the baking industry, we state that the studied method of water treatment will (i) accelerate the process of wheat grain swelling and producing flour from it, (ii) obtain a developed gluten matrix dough, and (iii) bakery products of high quality and long shelf life. Further research is recommended for active implementation of these studies in the food industry.

The use of scanning electron microscopy made it possible to visualize the food matrix, which led to a better understanding of the essence of the described processes. It was the fixation of the results at the microlevel that allowed us to form a hypothesis, which in the future should be confirmed by an additional array of experimental data.

## Figures and Tables

**Table 1:** The formulations of dough.

Ingredients, g	Wheat dough and bread samples						
	Control	a	b	c	d	f	g
Refined wheat flour (spring whitegrain wheat)	1000	1000	1000	1000	1000		
Refined wheat flour (winter whitegrain wheat)						1000	1000
Salt	15	15	15	15	15	15	15
Yeast	20	20	20	20	20	20	20
Drinking water	+						
Ultrasonic-treated water with a capacity of 252 W/l		+				+	
Ultrasonic-treated water with a capacity of 378 W/l			+				
Ultrasonic-treated water with a capacity of 504 W/l				+			
Ultrasonic-treated water with a capacity of 630 W/l					+		+

**Table 2:** Results of determining the specific volume and moisture content of bread.

Samples	Specific volume (ml g^−1^)	Moisture (%, wb^3^)
Control	3.66^1^ ± 0.09^2ab^	40.2 ± 0.5^cd^
a	3.24 ± 0.09^a^	40.8 ± 0.4^d^
b	3.34 ± 0.11^a^	41.4 ± 0.5^c^
c	3.89 ± 0.29^b^	41.6 ± 0.3^c^
d	4.20 ± 0.22^ab^	41.5 ± 0.2^c^
F	3.22 ± 0.12^a^	41.4 ± 0.3^c^
g	4.19 ± 0.12^ab^	41.6 ± 0.4^c^

^1^Means ±  ^2^standard deviation. Means in a row without a common superscript letter differ statistically (*p* < 0.05). ^3^ wb: wet basis.

## Data Availability

The data used and/or analyzed in the study are available from the corresponding author on reasonable request.
